# Proteome Analysis of Ground State Pluripotency

**DOI:** 10.1038/srep17985

**Published:** 2015-12-16

**Authors:** Sara Taleahmad, Mehdi Mirzaei, Lindsay M. Parker, Seyedeh-Nafiseh Hassani, Sepideh Mollamohammadi, Ali Sharifi-Zarchi, Paul A. Haynes, Hossein Baharvand, Ghasem Hosseini Salekdeh

**Affiliations:** 1 Department of Molecular Systems Biology, Cell Science Research Center, Royan Institute for Stem Cell Biology and Technology, ACECR, Tehran, Iran; 2 Department of Chemistry and Biomolecular sciences, Macquarie University, Sydney, NSW, 2109, Australia; 3 ARC Centre of Excellence for Nanoscale BioPhotonics (CNBP), Macquarie University, NSW, 2109, Sydney, Australia; 4 Department of Stem Cells and Developmental Biology, Cell Science Research Center, Royan Institute for Stem Cell Biology and Technology, ACECR, Tehran, Iran; 5 Department of Developmental Biology, University of Science and Culture, ACECR, Tehran, Iran; 6 Department of Systems Biology, Agricultural Biotechnology Research Institute of Iran, Karaj, Iran

## Abstract

The differentiation potential of pluripotent embryonic stem cells (ESCs) can be manipulated via serum and medium conditions for direct cellular development or to maintain a naïve ground state. The self-renewal state of ESCs can thus be induced by adding inhibitors of mitogen activated protein kinase (MAPK) and glycogen synthase kinase-3 (Gsk3), known as 2 inhibitors (2i) treatment. We have used a shotgun proteomics approach to investigate differences in protein expressions between 2i- and serum-grown mESCs. The results indicated that 164 proteins were significantly upregulated and 107 proteins downregulated in 2i-grown cells compared to serum. Protein pathways in 2i-grown cells with the highest enrichment were associated with glycolysis and gluconeogenesis. Protein pathways related to organ development were downregulated in 2i-grown cells. In serum-grown ESCs, protein pathways involved in integrin and focal adhesion, and signaling proteins involved in the actin cytoskeleton regulation were enriched. We observed a number of nuclear proteins which were mostly involved in self-renewal maintenance and were expressed at higher levels in 2i compared to serum - Dnmt1, Map2k1, Parp1, Xpo4, Eif3g, Smarca4/Brg1 and Smarcc1/Baf155. Collectively, the results provided an insight into the key protein pathways used by ESCs in the ground state or metastable conditions through 2i or serum culture medium, respectively.

Pluripotent embryonic stem cells (ESCs) are derived from the inner cell mass of blastocyst-stage embryos. These cells have a remarkable capacity to form differentiated cell types in culture, contingent upon extracellular signals. ESCs can be manipulated via serum and medium conditions for directed cellular development or alternatively to maintain a naïve ground state^1^. ESCs self-renewal success in mice is associated with bone morphogenetic protein 4 (BMP4)^2^ and/or leukemia inhibitory factor (LIF)^3^. BMP4 regulates the self-renewal of ESCs by inhibiting mitogen activated protein kinase (MAPK) pathways^2^ via SMAD proteins to suppress differentiation^4^. The LIF signaling pathway leads to phosphorylation of the transcription factor known as signal transducer and activator of transcription 3 (STAT3)^5^, a molecule which is critical in early embryonic development^6^.

Distinct transcriptome and epigenome profiles have been identified for ESCs grown in serum as opposed to a medium that contains inhibitors of MAPK and glycogen synthase kinase-3 (Gsk3), known as 2 inhibitors (2i) treatment^7^, suggesting that specific signaling pathways are required to support ESCs self-renewal. Although serum- and 2i-grown ESCs have similar potentials for differentiation, 2i-grown cells have lower expression of lineage affiliated genes, as well as bivalent domains which regulate transcriptional potential, and a higher expression of genes that regulate metabolic processes^7^. The key intracellular signaling pathways utilized by pluripotent ESCs that initiate differentiation or maintain a ground state remain to be identified at the proteome level.

In the current study, we described a quantitative proteomics screen for investigating differences in protein expressions of 2i- and serum-grown mouse ESCs by using label-free quantitative shotgun proteomics to identify and quantify proteins in complex protein mixtures in cellular lysates. We validated our proteomic findings with Western blot analysis by examining a number of proteins which significantly increased or decreased in the 2i-cultured ESCs compared to those grown in serum conditions. We additionally compared our proteomic findings to the previously reported transcriptome profile of 2i-grown cells in order to investigate whether additional post-translational modification pathways might contribute to ESC self-renewal capability.

## Results

### Morphology and characterization of mouse ESCs

The mouse ESCs propagated on in 2i/LIF and serum/LIF medium grow as compact colonies with a high nucleus-to-cytoplasm ratio and prominent nucleoli. These cells also retained expression of key mouse ESC markers including Oct-4 and SSEA1 (Fig. S1). However, as expected, cellular morphology and homogeneity of pluripotency-associated gene expression differed between the two growth conditions which was in line with previous report^1^. 2i ESCs were morphologically uniform and homogeneously expressed pluripotency-associated genes while serum ESCs were heterogeneous for both.

### Analysis of label-free shotgun proteomics

**Figure 1:**
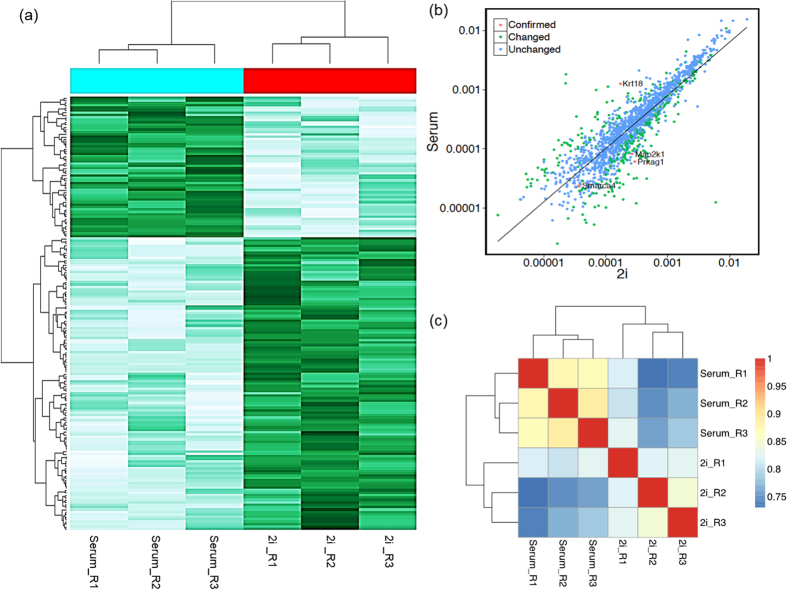
(**a**) Color map of the abundances. Reproducibly identified proteins in three biological replicates in 2i and serum samples that represent the logNSAF values for identified proteins, presented as logNSAF values of 2i samples on the x-axis and logNSAF values of serum samples on the y-axis. The dots are color coded, with light blue circles indicating that the logNSAF values are statistically unchanged between the two categories, while dark blue circles indicate those proteins with statistically significantly different logNSAF values between the two categories. Statistical significance is estimated using a student t-test on the log-transformed NSAF values from the original biological triplicates. The majority of data points are clustered along the diagonal, with the lower values closer to the origin. The further from the diagonal, the greater the degree of differential expression. (**b**) Expression pattern of proteins identified in 2i versus serum. (**c**) Correlation analysis of the samples. Rows and columns represent samples, and each square shows the Spearman correlation coefficients between two samples, indicated by its row and column. Hierarchical clustering of the samples is shown on the top and left sides.

A total of 1582 non-redundant proteins were reproducibly identified in 2i- and serum-grown samples. Data comparison showed that the majority of proteins (~83%) expressed at similar levels between the 2i- and serum-grown ESCs. The details of all reproducibly identified proteins are provided in Supplemental Table S1 online. The t-test analysis of proteins showed 271 differentially expressed proteins (p < 0.05), of which 164 proteins significantly upregulated and 107 significantly downregulated in ESCs treated with 2i compared to serum (Table S2). The Color map of the abundances of reproducibly identified proteins in 2i and serum samples is presented in Fig. 1a. Reproducibility of the data was confirmed by plotting the log NSAF values of the samples from 2i against serum (Fig. 1b). Spearman correlation coefficients between the two samples were analyzed. Hierarchical clustering of these is shown in Fig. 1c.

### Differential protein expression profiles between 2i and serum conditions

**Figure 2:**
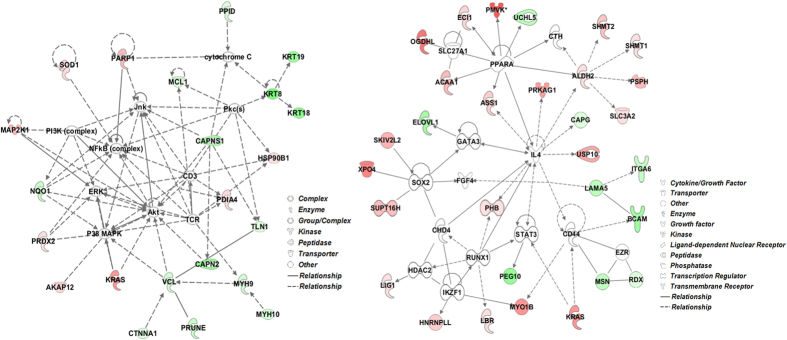
Protein network analysis using QIAGEN’s Ingenuity® Pathway Analysis (IPA®, QIAGEN Redwood City) software. Proteins identified in the dataset are highlighted in red or in green when they exhibit a higher or a lower content in 2i compared to serum. The intensity of the node color indicates the expression level or degree of regulation. Genes in uncolored notes were not differentially expressed in our experiment and were integrated into the computationally generated networks on the basis of the evidence stored in the IPA knowledge memory which indicated relevance to this network.

The key upregulated protein pathways following 2i treatment revealed by GO analysis (Table 1) were associated with metabolic and catabolic processes, and biosynthesis. Several proteins known to be involved in organ development - Myh9, Myh10, Hspg2, Flna and Lama5 (involved in muscle organ development), along with Anxa2, Sfn and epithelial cytokeratins (Krt8, Krt18 and Krt19) significantly downregulated following 2i treatment (Fig. 2a). Ingenuity pathway analysis was used to determine biological relationships among the 271 differentially expressed genes. The top networks, based on Fisher’s exact test, were associated with cellular movement, embryonic development and organ development, amino acid metabolism, cell cycle and lipid metabolism (Fig. 2a,b).

### Kyoto Encyclopedia of Genes and Genomes (KEGG) pathway enrichment analysis

**Figure 3:**
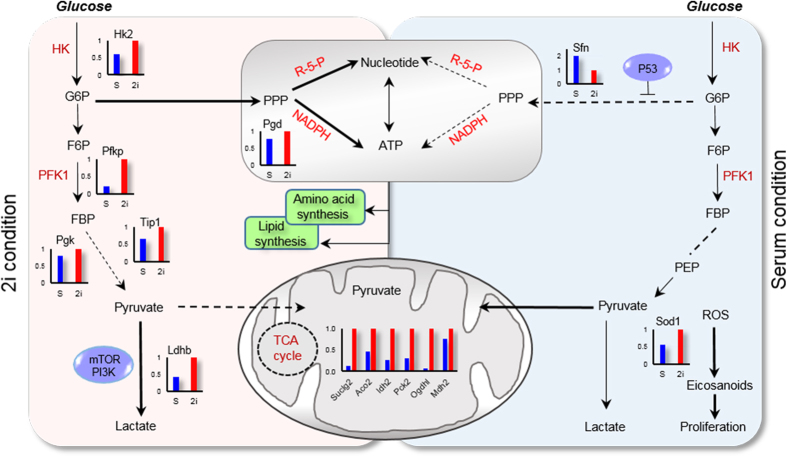
Key differences in metabolism between 2i and serum condition cells. (Left) Hexokinase (HK) and Phosphofructokinase 1 (PFK1) enzymes become activated in response to the sharply increased glycolytic flux, which resulted in increased flux into the pentose phosphate pathway (PPP) for nucleotide synthesis. mTOR and PI3K signaling pathways controlled glycolysis-regulating enzymes that included HK and LDH. (Right) Glycolytic flux and lactate production drop rapidly and flux into the PPP decreased. Increased ROS due to decreased in expression level of antioxidant enzyme (Sod1) promoted cell proliferation in the serum condition.

Kyoto Encyclopedia of Genes and Genomes (KEGG) pathway analysis identified glycolysis and gluconeogenesis associated pathways as the most significantly enriched pathways in 2i-grown cells (Fig. S2). Five key enzymes in the glycolysis pathway including hexokinase (HK), phosphofructokinase1 (PFK1), lactate dehydrogenase A (LDHA), triosephosphate isomerase 1 (TPI1), and phosphoglycerate kinase (PGK) significantly were upregulated in 2i-grown ESCs (Fig. 3). HK and PFK1 enzymes become activated in response to the sharply increased glycolytic flux, which resulted in increased flux into the pentose phosphate pathway (PPP) for nucleotide synthesis^8^. mTOR and PI3K signaling pathways upregulated in 2i which controlled the glycolysis-regulating enzymes, HK and LDH^9^. In mouse ESCs, mTOR has been defined as a key regulator of self-renewal. Expression levels of antioxidant enzymes such as Sod1 upregulated in 2i-grown ESCs, has suggested that reactive oxygen species (ROS) levels could be lower in this condition. ROS are critical mediators of cellular differentiation and tissue morphogenesis by oxidation of unsaturated lipid to form eicosanoid^10^.

**Figure 4:**
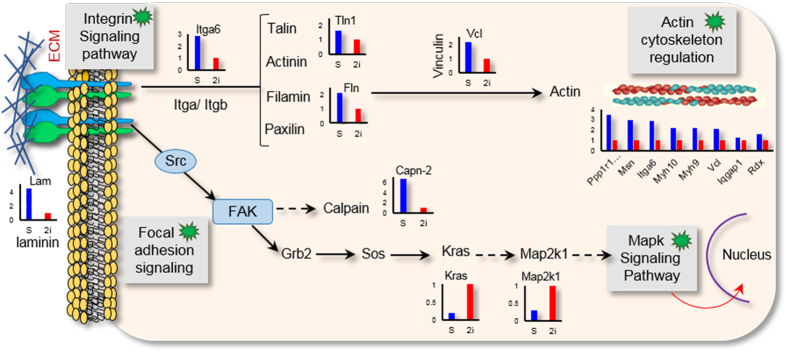
Key differences in the signaling pathway that was downregulated in 2i versus serum. Binding of integrin to ECM leads to integrin clustering on the cell membrane, formation of focal adhesion, and activation of signaling pathways, which depend on the specificity of integrin-ligand binding. Actin polymerization activated by an actin linkage that includes talin, vinculin and α-actinin; other signaling adaptors are also recruited to these complexes, including FAK and paxillin. Integrins are also associated with proteins involve non-receptor type kinases, such as Src, FAK, or ILK, which activate cellular transduction pathways, including the MAP kinase pathways.

Several metabolic pathways that included the citrate cycle, arginine and proline metabolism, fatty acid metabolism, and pyruvate metabolism were also upregulated following 2i treatment (Fig. S2). Interestingly, the Map2k1 and Kras proteins involved in the MAPK signaling pathway were upregulated in 2i-grown cells (Fig. 4). In serum-grown ESCs, we observed upregulation of integrin and focal adhesion signaling proteins involved in actin cytoskeleton regulation such as talin, vinculin, laminin and filamin (Fig. 4). Serum contains ECM components, such as laminin and fibronectin^11^, which negatively affect mouse ESCs self-renewal^12^. These components are adhesion molecules that express during the earliest stage in mouse development and are considered to be essential for proper development^13^.

### Differentially expressed nuclear proteins in 2i

We also compared the expression of nuclear proteins in 2i compared to serum treatment (Table 2). Among these, Xpo4 was upregulated by 6.8-fold. This protein is a member of the importin-ß family, which by binding of conserved peptide sequences in the MH2 domain of Smad3 is involved in nuclear transport of SMAD3 to the cytoplasm^14^. Sangel *et al*. have reported that by knockdown of Xpo4 the expression level of Sox2 decreased and selectively stimulated mouse ESCs differentiation towards a definitive endoderm while inhibiting NE differentiation^15^. Expression of poly (ADP-ribose) polymerase 1 (Parp1) was enhanced by 1.53-fold in 2i-grown ESCs. Parp1, a member of the Parp family of proteins, is a highly conserved DNA-binding protein abundant in the nucleus^16^. Parp1-catalyzed PARylation has been implicated in several processes, including enhancer binding, chromatin remodeling, insulation and co-regulation^17^. The abundance of Dnmt1 also increased by 1.9-fold. Dnmt1 is a major DNA methyltransferase responsible for maintaining methylation status during DNA replication. Nanog and Oct4 maintain self-renewal and the undifferentiated state in mesenchymal stem cells by regulating Dnmt1^18^. Smarca4/Brg1 and Smarcc1/Baf155 in 2i were significantly overexpressed in 2i compared to serum. Singhal *et al*. concluded that overexpression of Brg1 and Baf155 significantly enhanced OSKM-mediated reprogramming by achieving a euchromatic chromatin state and enhanced binding of reprogramming factors onto key reprogramming gene promoters^19^. The serine-threonine kinases, CDK2 and CDK7, were significantly more abundant in 2i-grown cells. Mouse ESCs use Thr-Gly metabolism to maintain their pluripotent epigenetic state^8^.

### Confirmation of shotgun proteomics results by Western blot analysis

**Figure 5:**
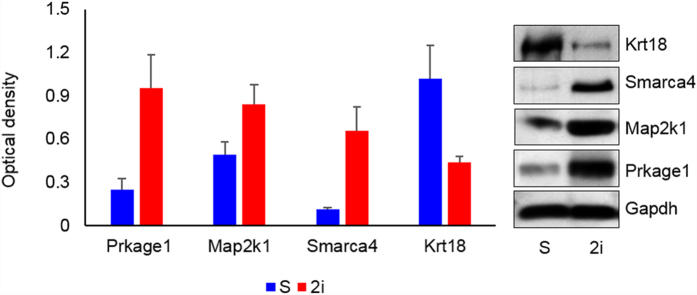
Western blot analysis. Fifty micrograms of protein from three biological replicates extracted from three independent replication of mouse ESCs cultured under 2i and serum conditions were subjected to SDS-PAGE followed by Western blotting. These proteins were analyzed with antibodies against Prkage1, Map2k1, Smarca4 and Krt18. Gapdh was used as the loading control. Protein bands were quantified by using ImageJ software. The y-axis represent the density of each band normalized to corresponding Gapdh band. Each column represents the mean ± SD from three experiments.

Our shotgun proteomics results were confirmed by Western blot analysis of four up or downregulated proteins including Prkage1, Map2k1, Smarca4 and Krt18 (Fig. 1b). These proteins were involved in biological processes that might be of interest for further functional analysis. The levels of Prkage1, Map2k1 and Smarca4 all increased in 2i whereas Krt18 level increased in the serum sample. There was very good agreement between the label-free quantitative shotgun proteomics data and the Western blot results as shown in Fig. 5. The expression level of Prkage1, Map2k1 and Krt18 were also confirmed by Western blot analysis in another mESC line, Royan B18 (Fig. S4).

### Correlated changes in the proteome and transcriptome of 2i treated embryonic stem cells

**Figure 6:**
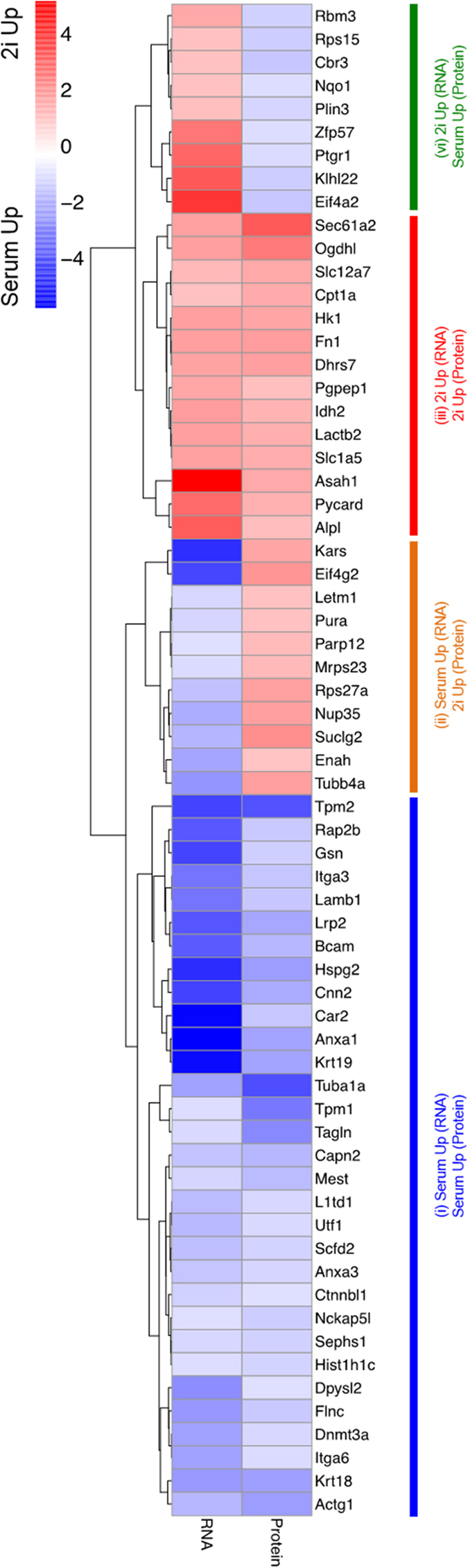
Comparison of transcriptome and proteome profiles of mESCs cultured in 2i and serum conditions. Each row of the heatmap represents a gene that has at least a 2-fold change between 2i and serum conditions in both the transcriptome and proteome profiles. Each cell of the heatmap indicates differential expression (RNA column) or protein concentration (Protein column) between 2i and serum, with red and blue colors indicative of higher expressions/ concentrations in the 2i and serum conditions, respectively. Hierarchical clustering of the genes is shown on the left side.

We compared our findings from 2i- or serum-grown ESCs with their previously reported corresponding mRNA levels^7^. Overall, the differentially expressed genes could be categorized in four different groups (Fig. 6): (i) both mRNA and protein upregulated in serum. This group comprised 31 genes and proteins mainly involved in actin binding and cytoskeletal organization. In this category, the biological process of muscle cell was upregulated and involved the Actg1, Capn2, Krt18, Krt19 and tpm1 proteins. Dnmt3a downregulated for both protein (3.5-fold) and mRNA levels under the 2i condition compared to serum. It has been demonstrated that the inhibition of Gsk3 and MEK by the 2i condition resulted in pronounced reduction in DNA methylation in ESCs and EGCs cell types. This was driven by Prdm14 and associated with downregulation of Dnmt3a and Dnmt3b^20^. It was demonstrated that the *de novo* methylation machinery including Dnmt3a, Dnm3b, and Dnmt3L was rapidly repressed in 2i, whereas maintenance genes such as Dnmt1 and Uhrf1 showed no change or were upregulated, respectively^21^. Habibi *et al*. showed that ESCs are globally hypomethylated in 2i condition compared to serum^22^. (ii) The expression levels of 11 proteins were upregulated, however the level of corresponding mRNAs downregulated in 2i compared to serum. Mitochondrial proteins were over-represented in this category. (iii) There were 14 mRNAs and their corresponding proteins were upregulated in the 2i sample. This category was enriched in genes involved in the citrate cycle (TCA cycle) KEGG pathway. (vi) Expression levels of 9 mRNAs increased in the 2i sample, but the level of the corresponding were downregulated. This included a number of proteins involved in translation and oxidation-reduction, such as Nqo1, Cbr3 and Ptgr1.

## Discussion

This study identified key protein pathways utilized by ESCs during self-renewal or differentiation. In the 2i sample, cells that promoted self-renewal, glycogenolysis and other metabolic pathways were most prevalent, whereas proteins that associated with organ were downregulated. This result suggested that serum-ESCs might be developmentally more advanced than 2i-ESCs^23^. In contrast, following serum conditions, the main protein pathways utilized were associated with integrin and focal adhesion signaling. Our results largely agreed with those described at the transcriptional level following 2i^7^ but suggested that a number of proteins underwent post-translational modifications.

Glycolysis provides biosynthetic precursors to support proliferation of pluripotent stem cells (PSCs). Although glycolysis is less efficient for energy production, this energy is produced faster and with lower ROS generation^24^. Higher amounts of energy are required to maintain specialized functions in different tissues upon cell differentiation whereas the lower proliferation capacity allow for a more efficient conversion of metabolic substrates into ATP^25^. Subsequent metabolic transition from glycolysis to oxidative phosphorylation (OXPHOS) is necessary for cellular differentiation. During iPSC development somatic cells switch metabolism from OXPHOS to enhanced glycolysis and become pluripotent when reprogrammed with pluripotent genes and/or small molecules^9^. The upregulation of mitochondrial enzymes (Ogdhl and Suclg2) related to oxidizing glucose from the TCA cycle is involved in amino acid synthesis. In most cells, glutamine is catabolized to α-ketoglutarate (αKG) to support TCA cycle anaplerosis. The elevated ratio of αKG/succinate in cells grown in 2i/L medium may play important roles in the regulation of chromatin structure^26^.

In order to initiate downstream signaling events, integrin signaling is dependent upon the non-receptor tyrosine kinase activities of focal adhesion kinase (FAK) and src proteins, as well as the adaptor protein functions of FAK, src and Shc^27^. The cytoplasmic tail of integrin serves as a binding site for talin and α-actinin, which subsequently recruit vinculin as a protein involved in anchoring F-actin to the membrane^28^. Signaling mediated by integrin-ECM interactions is integrated with the action of numerous other transmembrane signaling proteins to activate a wide range of cellular responses that include embryonic development, morphogenesis, cytoskeletal reorganization, cellular proliferation, migration, and survival^29^,^30^. Stem cell fate is regulated by soluble factors and interactions that involve cell-cell and cell-ECM contacts^31^. In this study, we have observed that proteins involved in focal adhesion and the integrin signaling pathway significantly downregulated in 2i-grown ESCs. Hayashi *et al*. suggested that ECM-integrin signaling inhibited mouse ESCs self-renewal by intensifying the activation of ERK1/2^12^. Previous studies cultured mouse ESCs on a strongly adhesive surface that would force them to spread. The results showed that these mouse ESCs downregulated pluripotency markers even when cultured in the presence of LIF^12^,^32^.

We have observed a number of nuclear proteins - Brg1, Baf155, Dnmt1, Map2k1, Parp1, Xpo4 and Eif3g, which expressed at higher levels in 2i compared to serum. These proteins are mostly involved in self-renewal and pluripotency maintenance. It has been shown that Brg1, an ATPase subunit of the SW1/SNF, plays a key role in proliferation and differentiation of cells^33^. SWI/SNF can regulate the interaction of histone modifying enzymes, transcription factors, and basal transcription machinery with chromatin^34^. In ESCs, knockdown of Smarca4 results in phenotypic changes indicative of differentiation, upregulation of differentiation genes and downregulation of self-renewal and pluripotency genes (e.g., Oct4, Sox2, Sall4, Rest)^35^. Brg1 may not only contribute to the repression of developmental genes but may also fine-tune the expression levels of ESC-specific genes, such as Oct4 and Sox2^35^,^36^. Parp1 is partly modulated by endogenous c-Myc and effectively enhances iPSC generation and helps to maintain a pluripotent state by post-translational regulation of protein PARylation^16^.

## Conclusion

Collectively, the results provided an insight into the key protein pathways utilized by ESCs in the ground state or metastable condition through 2i or serum culture medium, respectively. According to the quantitative shotgun proteomics approach, we observed marked differences between ESCs cultured in these two conditions. These quantitative evidences demonstrated that 2i could regulate protein expression, which would provide an excellent basis for further detailed study into a ground state signaling pathway. In line with the great concern for establishment of naïve human PSCs, quantitative proteomics of the ground state could provide an invaluable prototype for such interesting cells.

## Methods

### Culture of mouse embryonic stem cells

In this study we used a previously established mouse ESC line, Royan B20^37^. The cells were passaged simultaneously eight times in 2i/LIF and serum/LIF medium. All mouse ESCs were cultured without MEFs on 0.1% gelatin-coated plates (Sigma-Aldrich) using our previously described protocol^37^. Cells were routinely passaged every two days at a 1:3 ratio of cells passaged in each culture. The 2i treatment included the addition of Mek inhibitor PD0325901 (1 μM) and GSK3 inhibitor CHIR99021 (3 μM; Stemgent)^1^. Serum-free N2B27+2i or conventional mouse ESC serum media were used with LIF (Millipore). N2B27 medium consisted of DMEM/F12 (Invitrogen) and neurobasal (Invitrogen) at a 1:1 ratio, 1% N2 supplement (Invitrogen), 1% B27 supplement (Invitrogen), 2 mM L-glutamine (Invitrogen), 1% nonessential amino acids (Invitrogen), 100 U/ml penicillin, 100 mg/ml streptomycin (Invitrogen), 0.1 mM β-mercaptoethanol (Sigma-Aldrich), and 5 mg/mL bovine serum albumin (BSA; Sigma-Aldrich). Serum medium consisted of knockout Dulbecco’s modified Eagle’s medium (KoDMEM, Invitrogen), 15% fetal bovine serum (FBS, HyClone), 2 mM L-glutamine, 1% nonessential amino acids, 100 U/ml penicillin, 100 mg/ml streptomycin, and 0.1 mM β-mercaptoethanol.

### Protein extraction and separation by SDS-PAGE

Mouse ESCs samples, each containing at least 5 × 10^6^ cells, obtained from the three replicates of the 2i and serum groups were washed twice with 5 ml ice-cold PBS. The samples were then centrifuged at 450 × g for 5 min at 4 °C. After discarding the supernatant, 1 ml of the lysis buffer (Qiagen) containing 1 U benzonase nuclease and 10 μl of protease inhibitor (100×) was added to the cell precipitates followed by incubation at 4 °C on a rotary shaker for 5 min. The cells were then disrupted by three times of sonication on ice, each for 5 min (45 S pulse with 15 S rest intervals). The insoluble debris was then pelleted by centrifugation at 14000 × g at 4 °C for 10 min. The proteins contained in the supernatant were quantified by the Bradford Assay Kit (BioRad, Hercules, CA, USA) using BSA as standard. Moreover, the extracted proteins in sodium dodecyl sulfate (SDS) sample buffer (160 μg per well) were separated on 12% bis-tris polyacrylamide gels at 100 V for 1 h and were visualized using colloidal Coomassie blue. Finally, the gels were washed twice in water (10 min per wash) and the individual lanes were then cut into 12 slices of equal sizes from top to bottom.

### In-gel digestion by trypsin

Each stained gel piece was further reduced in size and was placed into individual wells of a 96-well plate. For destaining, the gel pieces were first briefly washed with 100 mM NH_4_HCO_3_, followed by washing with 200 μL of ACN (50%)/100 mM NH_4_HCO_3_ (50%) twice each 10 min. Then, the pieces were dehydrated with 100% ACN, air-dried, and reduced with 50 μL of 10 mM DTT/NH_4_HCO_3_ (50 mM) at 37 °C for 1 h. Finally, the samples were alkylated in the dark with 50 μL of 50 mM iodoacetamide/NH_4_HCO_3_ (50 mM) at room temperature for 1 h. After that, samples were briefly washed again with 100 mM NH_4_HCO_3_, 200 μL of ACN (50%)/100 mM NH_4_HCO_3_ (50%) for 10 min, dehydrated with 100% ACN and then air-dried. Finally, samples were digested with 20 μL of trypsin (12.5 ng/mL of 50 mM NH_4_HCO_3_) on ice for 30 min and then were left overnight at 37 °C. The resultant peptides were extracted twice with 30 μL of ACN (50%)/formic acid (2%), dried in a vacuum centrifuge, and reconstituted to 10 μL with 2% formic acid.

### Nanoflow liquid chromatography-tandem mass spectrometry

The digestate obtained from the SDS-PAGE gel slices were analyzed by nanoflow liquid chromatography tandem mass spectrometry (nanoLC-MS/MS) using an LTQ-XL ion-trap mass spectrometer (Thermo, Fremont, CA, USA). Reversed phase columns were packed in-house to approximately 7 cm (100 μmi.d.) using 100 Å, 5 μM Zorbax C18 resin (Agilent Technologies, Santa Clara, CA, USA) in a fused silica capillary with an integrated electrospray tip. A 1.8 kV electrospray voltage was applied via a liquid junction upstream of the C18 column. Samples were then injected into the C18 column using a Surveyor autosampler (Thermo, Fremont, CA, USA). The column was washed with buffer A [5% (v/v) ACN, 0.1% (v/v) formic acid] for 10 min at 1 μL/min before each loading. Peptides were subsequently eluted from the C18 column with 0–50% Buffer B [95% (v/v) ACN, 0.1% (v/v) formic acid] over 58 min at 500 nL per min followed by 50–95% Buffer B over 5 min at 500 nL per min. The column eluate was then directed into a nanospray ionization source of the mass spectrometer. Spectra were scanned over the range 400–1500 amu. Automated peak recognition, dynamic exclusion window set to 90s^38^ and tandem MS of the top six most intense precursor ions at 35% normalization collision energy were performed using Xcalibur™ software (version 2.06) (Thermo, Fremont, CA, USA).

### Protein identification

Raw files were converted to the mzXML format and were processed through Global Proteome Machine (GPM) software using version 2.1.1 of the X!Tandem algorithm which is available in the public domain at http://www.thegpm.org^39^,^40^. For each experiment, the 12 fractions were processed sequentially with output files for each individual fraction and a merged, non-redundant output file was generated for protein identifications with log (e) values less than −1. Tandem mass spectra were searched against the NCBI with the search parameters including MS and MS/MS tolerances of ±2 Da and ±0.2 Da, tolerance of 2 missed tryptic cleavages and K/R-P cleavages. Fixed modifications were set for carbamidomethylation of cysteine and variable modifications were set for oxidation of methionine.

### Quantitative proteomic analysis

Protein abundance data were computed based on normalized spectral abundance factors (NSAF) as previously described by Zybailov *et al*.^41^. For each protein k in sample i, the number of spectral counts which identified the protein by the estimated protein length was divided. The protein length was determined by dividing the molecular weight of the protein by the average amino acid molecular weight. The SpCk/Length values were normalized to the total by dividing by the sum (SpCk/Lengthk) over all proteins yielded NSAFi values for each sample i. When plotting or summarizing the overall protein abundance for a particular condition, the average of the NSAF values for all replicates as a measure of protein abundance was used. A spectral fraction of 0.5 was initially added to all spectral counts to compensate for null values and allowing for log transformation of the NSAF data prior to statistical analysis^42^.

### Statistical analysis

Several t-tests were performed to find the proteins that up- and downregulated between conditions. In each particular comparison, only the data set containing the proteins identified reproducibly, i.e. proteins present in all three biological replicates for at least one condition with the total spectral counts of minimum 5, were included. The 2-sample unpaired t-tests were run on log transformed NSAF data and proteins with a t-test p-value > 0.05 were considered to be differentially expressed. The resulting sets of up- and downregulated proteins were then annotated functionally.

### Functional annotation

The GI numbers of proteins were converted to Symbols and Entrez accession ID using the bioDBnet biological database network^43^. The lists of up- and downregulated proteins with relative GI ID for each comparison were then uploaded in DAVID (http://david.abcc.ncifcrf.gov/). The p-value and the Benjamini-Hochberg FDR were used to determine the significance of enrichment or overrepresentation of terms for each annotation [e.g., Gene Ontology (GO) biological process and KEGG pathway]. These proteins were also selected and reloaded into QIAGEN’s Ingenuity^®^ Pathway Analysis (IPA^®^, QIAGEN Redwood City, www.qiagen.com/ingenuity) for further analysis using gene symbols as identifiers, along with fold changes and adjusted p-values as observations with cutoff of 35 molecules per network and 25 network per analysis.

For GO categories of interest, NSAF abundance data were summed to achieve the overall protein abundance change over time for biological process categories. Then, GO annotation and relative protein abundance were plotted side by side for the up- and downregulated proteins for each of the comparison tests.

### Western blot analysis

Equal amount of proteins (50 μg) from three biological replicates were separated on a 12% SDS-PAGE gel and. The uniformity of the protein amount loaded on the gels was also visulized by staining SDS-PAGE gels using Coomassie Brilliant Blue (Fig. S3). For Western blot analysis, proteins were electrophoretically transferred onto polyvinylidine difluoride membranes (Bio-Rad). The blots were blocked with TBST (20 mM tris-HCl, pH 7.6, 150 mM NaCl, and 0.1%ween-20), containing 5% BSA, followed by incubation with primary antibody solution overnight at 4 °C. After washing with TBST, the membranes were incubated with horseradish peroxidase (HRP)-conjugated secondary antibody at room temperature for 1 h. Signals were detected with ECL substrate (GE) using Hyperfilm (GE). Supplemental Table S3 represents the primary and secondary antibodies used. Gapdh was used as the loading control. Protein bands were quantified by using ImageJ software. The volume of each band was analyzed using student’s t-test.

## Additional Information

**How to cite this article**: Taleahmad, S. *et al*. Proteome Analysis of Ground State Pluripotency. *Sci. Rep*. **5**, 17985; doi: 10.1038/srep17985 (2015).

## Supplementary Material

Supplementary Information

## Figures and Tables

**Table 1:** Biological process comparison of all differentially expressed proteins.

Biological process higher in 2i			Biological process higher in serum		
Term	Count	p-value	Term	Count	p-value
Metabolic process	101	3.14E-12	Organ development	17	0.047083
Cellular biosynthetic process	36	0.02085	Cellular localization	10	0.01278
Oxidation reduction	22	4.27E-07	Cell adhesion	9	0.01438
Homeostatic process	12	0.014633	Cell morphogenesis	7	0.031488
Glycolysis	8	1.06E-07	Cell motion	7	0.018195
Citrate cycle (TCA cycle)	6	4.57E-05	Cytoskeleton organization	7	0.029547
Glutamine family amino acid metabolic process	6	3.71E-05	Actin filament-based process	7	4.97E-04
Response to oxidative stress	5	9.25E-04	Muscle cell differentiation	5	0.029134

**Table 2:** List of nuclear proteins highly expressed in 2i (at least 1.5-fold change).

Identifier	Symbol	Gene name	Ratio	p-value
124107596	Prkag1, 5	AMP-activated protein kinase	4.8	0.001
9506945	Pabpn1	Polyadenylate-binding protein 2	4.7	0.03
21312352	Skiv2l2	Superkiller viralicidic activity 2-like 2	3.4	0.04
6678794	Map2k1	Dual specificity mitogen-activated protein kinase 1	3.3	0.003
36031035	Smc3	Structural maintenance of chromosomes protein 3	3	0.03
20806109	Parp1	Poly [ADP-ribose] polymerase 1	2.9	0.02
120407050	Nop58	Nuclear protein 58	2.3	0.002
134031976	Lrpprc	Leucine-rich PPR motif-containing protein	2.2	0.01
110625924	Rfc5	Replication factor C subunit 2	2.2	0.001
100815493	Wapal	Wings apart-like protein homolog	2	0.04
112421097	Smarcc1	SWI/SNF complex subunit	1.9	0.05
327180732	Dnmt1	DNA (cytosine-5)-methyltransferase 1	1.9	0.04
108773813	Nup85	Nuclear pore complex protein Nup85	1.9	0.01
6754744	Msh6	DNA mismatch repair protein	1.8	0.007
34556205	Cdk2	Cyclin-dependent kinase 2 isoform 1	1.6	0.04
160333726	Cdk7	Cyclin-dependent kinase 7	1.6	0.04
133892807	Lig1	DNA ligase 1	1.5	0.04
291463271	Smarca4	Transcription activator BRG1	1.5	0.005
