# Supplementary material for: Proteome Analysis of Ground State Pluripotency
Source: Sci Rep. 2015 Dec 16;5:17985. doi: 10.1038/srep17985 (PMC4680864; doi:10.1038/srep17985)
Supplement: Supplementary Information [file srep17985-s1.pdf]

Proteome Analysis of Ground State Pluripotency

Sara Taleahmad[1 †], Mehdi Mirzaei[2 †], Lindsay Parker[2], Seyedeh-Nafiseh Hassani[3], Sepideh Mollamohammadi[3], Ali Sharifi-Zarchi[3], Paul A. Haynes[2] , Hossein Baharvand[3,4,*] and Ghasem Hosseini Salekdeh[1,5,*]

1. Department of Molecular Systems Biology at Cell Science Research Center, Royan Institute for Stem Cell Biology and Technology, ACECR, Tehran, Iran
2. Department of Chemistry and Biomolecular sciences, Macquarie University, Sydney, NSW, 2109, Australia
3. Department of Stem Cells and Developmental Biology at Cell Science Research Center, Royan Institute for Stem Cell Biology and Technology, ACECR, Tehran, Iran
4. Department of Developmental Biology, University of Science and Culture, ACECR, Tehran, Iran
5. Department of Systems Biology, Agricultural Biotechnology Research Institute of Iran, Karaj, Iran

[†] Equally contributing authors

***Corresponding authors:**

Ghasem Hosseini Salekdeh, Department of Molecular Systems Biology at Cell Science Research Center, Royan Institute for Stem Cell Biology and Technology, ACECR, Tehran, Iran. Tel: +98 21 22306485, Fax: +98 21 23562507, Email: Salekdeh@RoyanInstitute.org

Or

Hossein Baharvand, Department of Stem Cells and Developmental Biology at Cell Science Research Center, Royan Institute for Stem Cell Biology and Technology, ACECR, P.O. Box 19395-4644, Tehran, Iran. Tel: +98 21 22306485, Fax: +98 21 23562507, Email: Baharvand@RoyanInstitute.org

Additional Information

Supplemental Figure S1. Characteristics of the mouse ES cells cultivated in serum and 2i condition. Phase contrast and immunofluorescence labeling for Oct4and SSEA-1, counterstained for DAPI are shown.

Supplemental Figure S2. Significantly enriched KEGG pathway analysis of 2i and serum samples. The x-axis represent the number of proteins which involved in KEGG pathway signaling. The pathways having enrichment ($p<0.01$) are presented.

Supplemental Figure S3. The SDS-PAGE gel image of proteins from 2i- and serum-grown ESCs. M; Molecular weight marker (MagicMark™ XP), Lane 1-3; 3 replicates of serum-grown ESCs protein, Lane 4-6; 3 replicates of 2i-grown ESCs protein.

Supplemental Figure S4. Western blot analysis. Fifty micrograms of protein from three biological replicates extracted from three independent replication of mESC line (Royan B18) cultured under 2i and serum conditions were subjected to SDS-PAGE followed by Western blotting. These proteins were analyzed with antibodies against Prkage1, Map2k1 and Krt18. The y-axis represent the density of each band normalized to corresponding Gapdh band. Each column represents the mean ± SD from three experiments.

Supplemental Table S1. The complete set of 1582 proteins identified reproducibly from cells cultured under 2i and serum conditions in this study, including numbers of peptides assigned to each protein in each replicate experiment. Also included are protein identification data from individual replicates with measured and corrected percent coverage values for each protein.

Supplemental Table S2. List of proteins up or down regulated in 2i-grown cells versus serum.

Supplemental Table S3. Primary and secondary antibodies used for Western blotting.

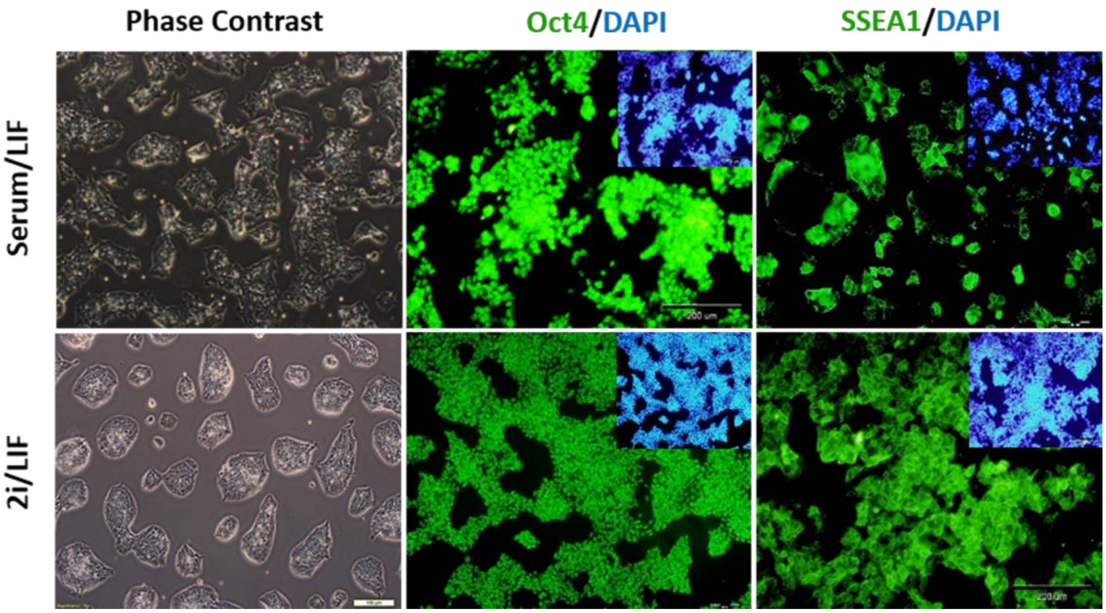

Figure S1.

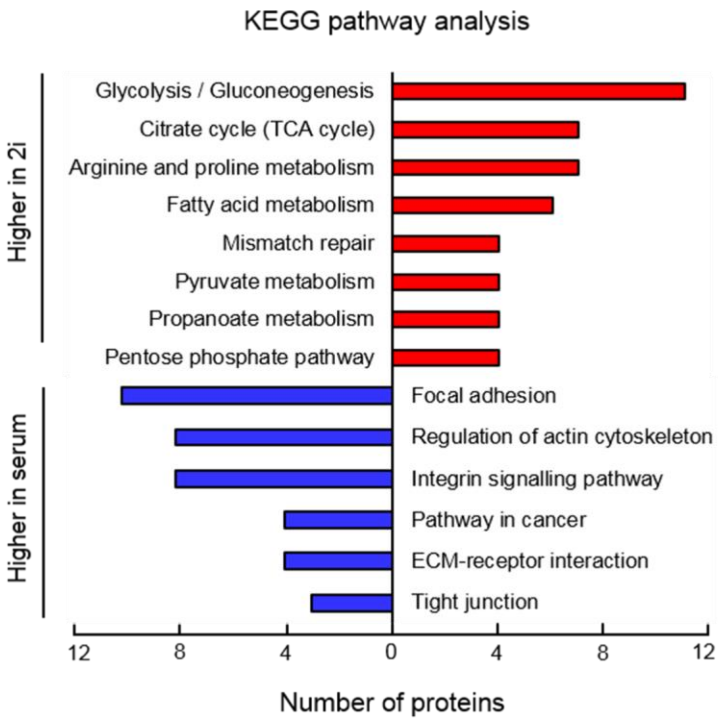
KEGG pathway analysis
Higher in 2i
Glycolysis / Gluconeogenesis
Citrate cycle (TCA cycle)
Arginine and proline metabolism
Fatty acid metabolism
Mismatch repair
Pyruvate metabolism
Propanoate metabolism
Pentose phosphate pathway
Higher in serum
Focal adhesion
Regulation of actin cytoskeleton
Integrin signalling pathway
Pathway in cancer
ECM-receptor interaction
Tight junction
12
8
4
0
4
8
12
Number of proteins

Figure S2.

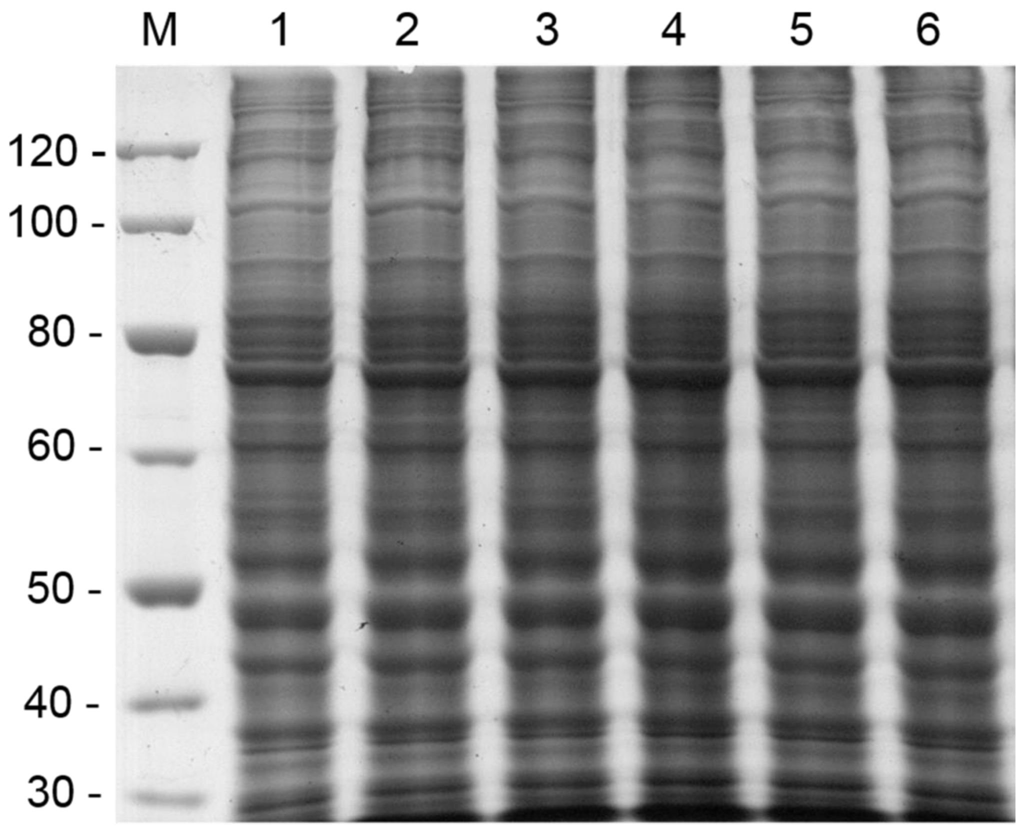

Figure S3.

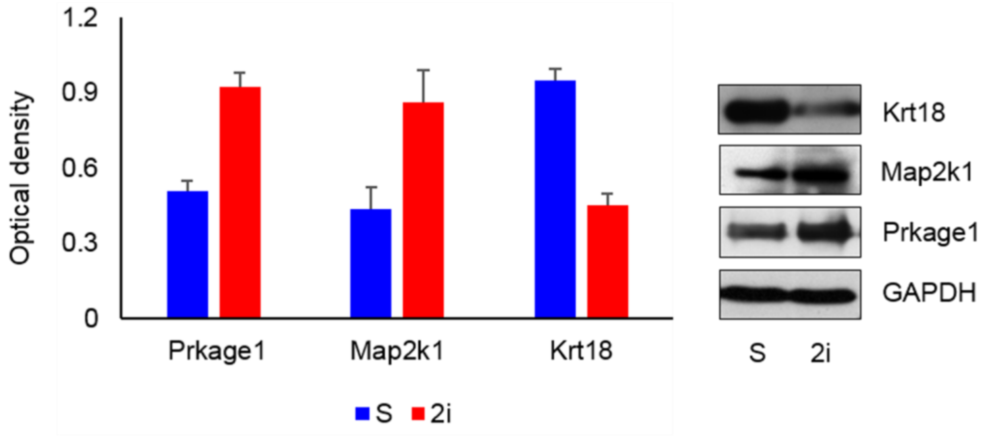
1.2
0.9
0.6
0.3
0
Optical density
Prkage1
Map2k1
Krt18
S
2i
Krt18
Map2k1
Prkage1
GAPDH
S
2i

Figure S4.

Supplemental Table S3. Primary and secondary antibodies used for Western blotting.

Target	Conc.	MW (kDa)	Company, Cat. No.
Primary antibodies			
Prkage1	1:100	37	Cell signaling, 4187s
Map2k1	1:1000	43	Sigma-Aldrich, SAB2107602
Smarca4	1:1000	181	Cell signaling, 3508s
Krt18	1:200	47	EMD Millipore, 04-586
GAPDH	1:4000	36	Sigma-Aldrich, G9545
Secondary antibodies			
Mouse IgG-HRP	1:50000		Sigma-Aldrich, A0168
Rabbit IgG-HRP	1:50000		Sigma-Aldrich, A0545
